# Efficacy and Safety of *Houttuynia* Eye Drops Atomization Treatment for Meibomian Gland Dysfunction-Related Dry Eye Disease: A Randomized, Double-Blinded, Placebo-Controlled Clinical Trial

**DOI:** 10.3390/jcm9124022

**Published:** 2020-12-12

**Authors:** Zhaolin Liu, Ming Jin, Ying Li, Jun Liu, Xianghua Xiao, Hongsheng Bi, Zhiqiang Pan, Huijun Shi, Xiaofeng Xie, Minglian Zhang, Xuemin Gao, Lei Li, Weijie Ouyang, Liying Tang, Jieli Wu, Yiran Yang, Jiaoyue Hu, Zuguo Liu

**Affiliations:** 1 Department of Ophthalmology, Xiang’an Hospital of Xiamen University, Fujian Provincial Key Laboratory of Ophthalmology and Visual Science, Xiamen 361102, China; zhaolin@stu.xmu.edu.cn (Z.L.); 24520190154789@stu.xmu.edu.cn (W.O.); liyingtang@stu.xmu.edu.cn (L.T.); 24520180155744@stu.xmu.edu.cn (J.W.); yangyr1026@stu.xmu.edu.cn (Y.Y.); jiaoyuehu@xmu.edu.cn (J.H.); 2 Eye Institute of Xiamen University, School of Medicine, Xiamen University, Xiamen 361102, China; 3 Department of Ocular Surface, Xiamen University Affiliated Xiamen Eye Center, Xiamen 361102, China; 4 Department of Ophthalmology, China-Japan Friendship Hospital, Beijing100029, China; jinming57@163.com; 5 Department of Ophthalmology, Peking Union Medical College Hospital, Beijing 100006, China; liyingpumch@126.com; 6 Department of Ophthalmology, Shenzhen Eye Hospital, Shenzhen 518040, China; jiu28@126.com; 7 Department of Ophthalmology, Xi’an First Hospital, Shaanxi Eye Research Institute, Shaanxi 710001, China; xianghuaxiao@yeah.net; 8 Department of Ophthalmology, Shandong University of Traditional Chinese Medicine Affiliated Eye Hospital, Jinan 250004, China; hongshengbi@126.com (H.B.); yankeboshi@126.com (X.X.); 9 Department of Ophthalmology, Capital Medical University Affiliated Beijing Tongren Hospital, Beijing 100730, China; panyj0526@sina.com; 10 Department of Ophthalmology, Hebei Eye Hospital, Xingtai 054001, China; 15631901079@163.com (H.S.); zhmlyk@sohu.com (M.Z.); 11 Technical Center for Drug Research and Evaluation of China Association of Traditional Chinese Medicine, Beijing 100061, China; xuemingao2011@163.com (X.G.); cptlilei@sina.com (L.L.)

**Keywords:** *Houttuynia*, ultrasonic atomization, meibomian gland dysfunction, dry eye

## Abstract

Purpose: To evaluate the efficacy and safety of *Houttuynia* eye drops (a Chinese traditional medicine) atomization treatment in meibomian gland dysfunction (MGD)-related dry eye disease (DED) patients. Methods: A total of 240 eligible patients diagnosed with MGD-related DED were assigned either *Houttuynia* eye drops or placebo for atomization once daily for four weeks in a multi-center, randomized, double-blind, placebo-controlled clinical study. Primary outcome evaluations used included eye symptom score (using the Chinese Dry Eye Questionnaire), meibum quality, and tear break-up time (TBUT), while safety evaluations included adverse events (AEs), visual acuity, and intraocular pressure monitoring. Indicators were measured at baseline as well as one week, two weeks, and four weeks after treatment. Results: Primary outcome measures of the *Houttuynia* group were improved compared with their placebo counterparts following four-week treatment. Eye symptom scores were significantly reduced relative to the baseline in the *Houttuynia* group (mean ± standard error of the mean, 9.00 ± 0.61) compared with the placebo group (6.29 ± 0.55; *p* = 0.0018). Reduction in meibum quality score in the *Houttuynia* group (0.91 ± 0.10) was also significantly higher compared with the placebo group (0.57 ± 0.10; *p* = 0.0091), while TBUT in the treatment group (6.30 ± 0.22) was also longer than in the latter (5.60 ± 0.24; *p* = 0.0192). No medication-related adverse events were observed. Conclusions: Atomization treatment with *Houttuynia* eye drops is both clinically and statistically effective for the treatment of mild to moderate MGD-related DED patients. This approach is generally safe and was tolerated well by patients.

## 1. Introduction

TFOS DEWS II [1] has defined dry eye is a multifactorial disease of the ocular surface characterized by a loss of homeostasis of the tear film and accompanied by ocular symptoms, in which tear film instability and hyperosmolarity, ocular surface inflammation and damage, and neurosensory abnormalities play etiological roles. The prevalence of dry eye disease (DED) is currently between 5% and 50% globally and particularly high in Asian countries [2,3,4]. The causes of DED are very complex, including age, systemic immune disease, visual display terminal, and meibomian gland dysfunction (MGD) [1]. MGD is one of the most common causes of DED overall and is also the main underlying factor leading to evaporative dry eye (EDE). Thus, DED caused by MGD is also known as MGD-related DED. Epidemiological investigations showed that 80% of DED patients have either developed this condition from MGD or are also suffering from this affliction [3]. Prevalence rates of MGD-related DED in Asian populations over the age of 40 range between 46.2% and 69.3% [4]. It is clear that MGD-related DED is characterized by terminal duct obstructions and/or qualitative/quantitative changes in glandular secretions; this latter effect reduces lipid secretion to tear film and increases evaporation, resulting in tear hyperosmolarity. These tear changes further induce inflammatory cascade reactions which then cause a series of clinical symptoms. Changes in tear fluid also aggravate destruction of the eyelid margin and meibomian gland, conditions which then go on to develop into a vicious cycle [5,6,7].

A variety of methods are utilized for the treatment of MGD-related DED, including physical therapies such as meibomian gland massage, intraductal meibomian gland probing, lipiflow, intense pulsed light, and local anti-inflammatory drugs. Although these approaches are all effective to a certain extent, problems remain including the inconvenience of application, high financial costs, insignificant effects, and some side effects [5,8]. It is therefore necessary to consider ways to explore new therapies that are safe, effective, and convenient in order to improve MGD-related DED treatment.

Traditional medicine has often been used for the clinical treatment of DED in China, and a great deal of documentation supports the fact that this approach achieves positive therapeutic effects [9]. Ultrasonic atomization is a method that destroys liquid surface tension and atomizes droplets into fine molecules via ultrasonic vibration. It is the most commonly utilized ophthalmological method in Chinese traditional medicine for DED treatment. This therapy for 20 min enables the drug to fully contact and penetrate the ocular surface and take effect. Stimulation of an atomized steam can promote blood and lymph circulation in eyelid tissue and thus enhance the discharge of meibomian gland secretions [10]. The majority of Chinese hospitals possess specialized ultrasonic atomization units and traditional medicine is the most commonly applied treatment. Although several studies have evaluated the therapeutic effects of ultrasonic nebulization for the treatment of MGD, DED, and MGD-related DED [10,11,12], clear clinical evidence is lacking from a standardized multicenter, double-blind, randomized, controlled clinical study.

The plant *Houttuynia cordata* is used in traditional Chinese medicine because it induces anti-inflammatory effects. This therapy mainly reduces the levels of inflammation-related cytokines and chemokines by inhibiting the Nuclear Factor Kappa-B (NF-κB)/Mitogen-Activated Protein Kinase (MAPK) pathway, leading to anti-inflammatory effects [13,14,15]. Thus, *Houttuynia* eye drops have been widely used across China for the treatment of DED. A number of non-double-blind, non-randomized, single-center small sample studies have shown that ultrasonic atomization using *Houttuynia* eye drops is both safe and effective for treating dry eye [16,17]. Therefore, building on this earlier work and in order to evaluate the specific therapeutic effects and safety of ultrasonic-atomized *Houttuynia* eye drops for the treatment of MGD-related DED, the China Association of Traditional Chinese Medicine (CATCM) organized the novel multi-center, randomized, double-blind, placebo-controlled trial reported in this study. So far, this is the first multicenter, randomized, double-blind, placebo-controlled study to evaluate the efficacy and safety of traditional Chinese medicine for the treatment of DED.

## 2. Methods

This randomized, double-blind, placebo-controlled, multi-center study was conducted at eight centers across China (Table S1) between 31 October 2018 and 29 January 2019. The design of this clinical trial complied with local laws and regulations and was developed in accordance with accepted standards for Good Clinical Practice of Pharmaceutical Products (2003) [18], Provisions of Drug Registration (2007) [19], and the Declaration of Helsinki. Data acquisition and analysis were performed in compliance with protocols approved by the Ethical Committee of Xiamen University and China-Japan Friendship Hospital (*ethical approval number 2018-97-K69*) and were prospectively registered on the Chinese Clinical Trial Registry www.chictr.org.cn (ChiCTR1800018611). All subjects signed informed consent forms prior to study initiation.

### 2.1. Study Patients

A total of 120 cases were included in the experimental *Houttuynia* group and the control placebo group, respectively, by applying the 1:1 design principle. Patients needed to meet the following inclusion criteria. First, subjects were required to be aged between 18 and 70 years and had to be diagnosed with MGD-related DED. Diagnostic criteria for MGD were according to the “Expert consensus of diagnosis and treatment of meibomian gland dysfunction in China (2017)” [20]. People who had eye symptoms, combined with abnormal eyelid margin and meibomian gland opening, or abnormal meibum secretion, were diagnosed as MGD. Second, diagnostic criteria for DED were according to the “Expert consensus on clinical diagnosis and treatment of dry eye (2013)” [21]. The following examination test criteria were required: 2 mm/5 min < Schirmer I test ≤ 10 mm/5 min, 0 s < TBUT ≤ 10 s, and corneal fluorescence staining point numbers < 10 points. That is, only patients with mild or moderate dry eye were included because severe dry eye generally needs to be combined with other treatments. Third, patients had to not use, or had to have stopped using, artificial tears for more than seven days. We chose just one eye from each subject for observations; if both eyes met the inclusion criteria, the more severely affected eye was used, while if the condition in both eyes was identical, the right one was used.

Patients were excluded if they had conditions including additional eye diseases such as macular degeneration, glaucoma, keratitis, and retinal vascular embolism, as well as lacrimal passage obstructions, prominent exophthalmos, and severe corneal decompensation. Patients were also excluded if they had undergone eye surgery less than half a year ago, if they had a history of wearing corneal contact lens up to one week prior to enrollment, or if they had suffered from other serious primary diseases including those of the cardiovascular, cerebrovascular, liver, kidney, or hematopoietic systems. Patients were excluded from this trial if they had an allergic constitution or were allergic to experimental drug ingredients. Exclusion also took place if patients had participated in other clinical trials within the last three months, or if individuals were unable to cooperate or were mentally ill.

### 2.2. Study Design

A double-blind design was used in this study. Thus, the 1:1 ratio between experimental treatment and control groups was used to generate random codes using the statistical software SAS9.4 (SAS Institute Inc., Cary, NC, USA), applying the block randomization method. Subjects were then assigned a medication box labeled with a serial number which contained all medications for the treatment duration. The experimental group used commercially available *Houttuynia* eye drops (specification: 8 mL; national drug approval no. Z20010110; Sichuan Shenghe Pharmaceutical Co. Ltd., China). The exact composition of Houttuynia eye drops was 2-undecanone (C_11_H_22_O) at the concentration of 9.8 µg/mL, and 2-undecanone was the main effective component of the Houttuynia cordata extract [14,15]. Meanwhile, the control group was treated with a placebo agent (Sichuan Shenghe Pharmaceutical Co. Ltd., China), and the placebo was a 0.72% (g/mL) sodium chloride solution with a pH value of 6.7 and osmotic pressure of 237 mOsmol/kg. Patients were also equipped with handheld ultrasonic nebulizers (product number: HL100A, Yuwell, Jiangsu, China) (Figure S1). The medication application method used in this study was atomizing each eye for 20 min per day with the ultrasonic nebulizer held in front of each eye. The atomizing tube was placed 5–10 cm away from the eye, and the patient kept their eyes wide open, staring in all directions intermittently to ensure that the atomizing agent fully touched the conjunctival sac. Patients used 20 mL houttuynia cordata eye drops or placebo at a time. The temperature of an ultrasonic-atomized aerosol is very close to room temperature, making the therapy induce slight irritation. The treatment course was four weeks with visit points at time zero (baseline) and then after one week, two weeks, and four weeks; treatment efficacy and safety assessments were carried out on-site during these visits. All tests were carried out using the same equipment type. The tester and the statistician were both unaware of patient groupings.

### 2.3. Outcome Measures

Primary outcomes evaluated included eye symptom score (using the Chinese Dry Eye Questionnaire, which is a dry eye questionnaire that conforms to the use habits of Chinese patients; the clinical diagnosis of dry eye in Chinese patients showed better diagnostic value than the OSDI questionnaire) [22] (Table S2), meibum quality, and tear break-up time (TBUT). Secondary outcomes evaluated included meibum expressibility, meibomian gland dropout, eyelid margin change, conjunctival congestion, corneal staining, and the Schirmer I test [23].

Sodium fluorescein TBUT was measured using a commercial fluorescein strip which was moistened and applied to the lower eyelid conjunctiva. One minute after application, patients were then asked to blink three times and to hold their eyes open. The time between the eyes open and the appearance of the first dark spot on the precorneal film was then measured using a slit lamp with cobalt blue illumination and a yellow-barrier filter. This was repeated three times before an average for each eye was recorded [21].

Meibomian gland functionality was assessed by applying digital pressure to the central (nasal, temporal) third of the lower/upper lids in order to determine MGD extent and severity (i.e., expressibility and meibum quality). Scoring values for meibum quality were as follows: 0, clear (normal); 1, cloudy; 2, cloudy with particles; and 3, inspissated (like toothpaste). Values were recorded for the highest grade encountered from any expressed glands, encompassing a range between 0 and 3. Similarly, meibum expressibility scoring criteria were as follows: 0, all glands expressed; 1, 3–4 glands expressed; 2, 1–2 glands expressed; and 3, no glands expressed. Meibum expressibility was scored based on five glands from the nasal side, while values for the middle and temporal sides of the lower eyelid were also recorded. Total scores for these three parts were recorded at the same time, across a range between 0 and 9 [23].

The eyelid margin change scoring criteria used follow standards set by the international MGD consensus group, while the presence or absence of lid abnormalities were scored via irregularity of the lid margin, lid margin vascular engorgement including plugging of the meibomian orifices, and anterior or retroplacement of the MCJ. These were either scored as present (1) or absent (0), across a range between 0 and 4 [23].

Meibomian gland dropout scoring criteria refer to the loss of acinar tissue detected by meibography. Non-contact meibography using a standard infrared video security camera was examined in patients. Scoring values for meibomian gland dropout were as follows: 0, no gland dropout; 1, between 1% and 33%; 2, between 34% and 66%; and 3, ≥67% dropout. The percentage of partial, or total, gland dropout of the lower lid was recorded across a range between 0 and 3 [23].

The MGD staging was determined according to the score of meibum quality, eyelid margin change, meibomian gland expressibility (middle meibomian gland expression score), and dropout. The higher these index scores were, the more serious the MGD was [20].

The conjunctival congestion scoring criteria used here follow the classification outlined by the Cornea and Contact Lens Research Unit, as follows: 0, no hyperemia; 1, mild hyperemia; 2, moderate hyperemia; and 3, severe hyperemia [24].

The corneal staining scoring criteria involved fluorescein staining that was examined two minutes subsequent to the instillation of sodium fluorescein at the slit lamp using cobalt blue illumination and a yellow-barrier filter. The 12-points method was used to assess the staining degree. Each of the four quadrants of the cornea on a scale ranged between 0 and 3: 0, no staining; 1, between one and 30 dots stained; 2, >30 dots stained without fusion; and 3, corneal diffuse or coalescent punctate staining, corneal filaments, and an ulcer. Scores for the four quadrants were then summed to attain a total score for each eye across a range between 0 and 12 [21].

The Schirmer I test was performed on unanesthetized patients by placing a strip in each eye and recording the wetted length (mm) after five minutes [21].

Eye tolerance, irritant symptoms, and treatment satisfaction values were also incorporated in this analysis.

The safety evaluations included adverse events (AEs), routine eye examinations (including monitoring of uncorrected/corrected visual acuity and intraocular pressure (IOP)), vital signs, and laboratory indicators (including blood and urine routine, liver and kidney function) which were selected according to the requirements of each hospital and the condition of subjects. Artificial tears, anti-inflammatory eye drops, and physical therapies such as eyelid cleaning, the use of hot compresses, meibomian gland massages, acupuncture, and silicone eye masks were prohibited. The comorbidity of patients and the use of any drug or treatment for the comorbidity was recorded, including the drug name (or other treatment), dosage, number, and time of use. If a prohibited drug or treatment was used, the subjects were required to discontinue the study.

### 2.4. Statistical Methods

A descriptive quantitative statistical analysis was performed encompassing the number of cases, mean, standard error of the mean (SEM), and 95% confidence intervals (CIs). A group *t*-test or a Wilcoxon rank-sum test was then used to make comparisons between groups and covariance analysis was also performed. In the case of qualitative data, a descriptive statistical analysis was carried out on the number of cases in various categories and their percentages. Thus, the chi-squared test alongside the Fisher exact test was used to compare enumerated data between the different treatment groups, while a Wilcoxon rank-sum test was used for comparison amongst treatment groups. A CMH-x^2^ test was used when the center or other factors were considered, while the relationship between a reduction in eye symptom score after four weeks of treatment and baseline scores was analyzed using Spearman’s correlation coefficient. The reduction in eyelid margin change score vs. the baseline was also examined using Spearman’s correlation coefficient, following the intention-to-treat principle (ITT). The latest observation data were carried forward to the final test result in each case to determine missing values for the main efficacy evaluation index (the last observation carried forward (LOCF) method). The post hoc subgroups were defined according to baseline severity of symptoms (eye symptoms score ≥15 vs. <15) and severity of signs (meibum quality score 0–3, middle meibomian gland expression score 0–3, eyelid margin change score 0–4, conjunctival congestion score 0–2, corneal staining score 0, 1, >1). Tests of interaction were used to evaluate whether the effect of atomization treatment with Houttuynia eye drops and placebo differed among subgroups. The software packages SAS9.4 (SAS Institute Inc., Cary, NC, USA) and GraphPad Prism version 8.2.1 (GraphPad Software, San Diego, CA, USA) were used for all statistical calculations. All tests were two-sided, and *p* < 0.05 was considered significant.

## 3. Results

**Figure 1:**
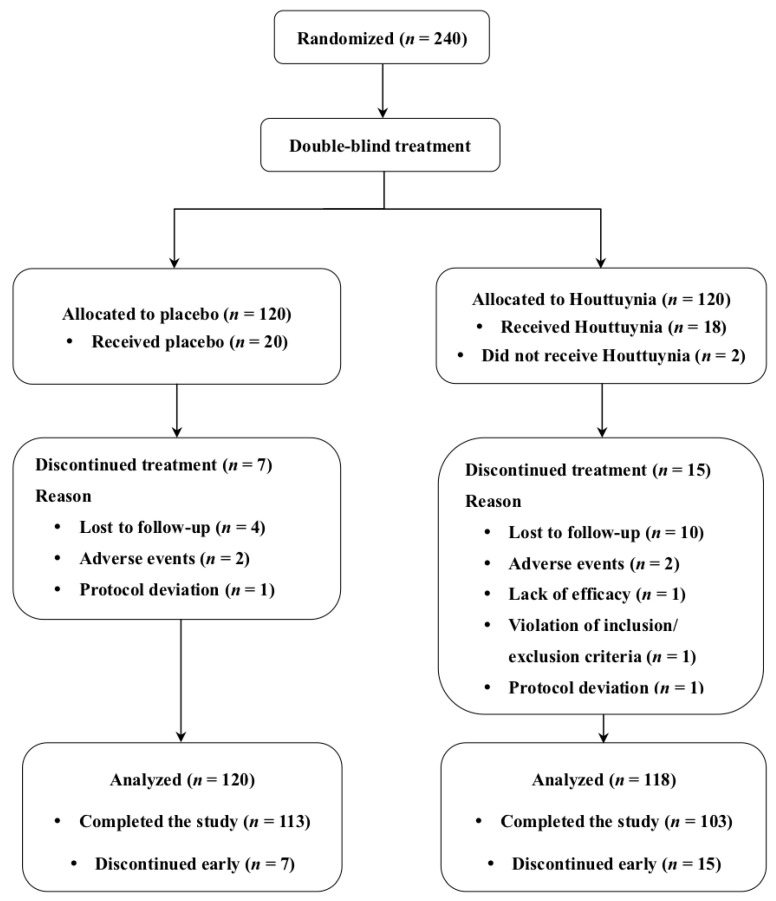
Subject flow chart.

A total of 240 selected patients were randomized for double-blind treatment. Two patients in the Houttuynia group did not take medication, thus 120 and 118 patients in the placebo and Houttuynia groups, respectively, were analyzed. A total of 113 and 103 patients completed all study visits, respectively; this means that seven and 13 patients dropped out from each group for various reasons (Figure 1). The data in Table S3 demonstrate patient clinical characteristics at baseline. These data show no significant differences in demographic characteristics between the placebo and Houttuynia groups, comprising about 75% of women with an average age of about 38 years. Results also show no statistical differences in the data at baseline between the two groups in terms of ocular symptoms as well as in signs of MGD and DED.

### 3.1. Efficacy Evaluation

#### 3.1.1. Primary Outcomes

**Figure 2:**
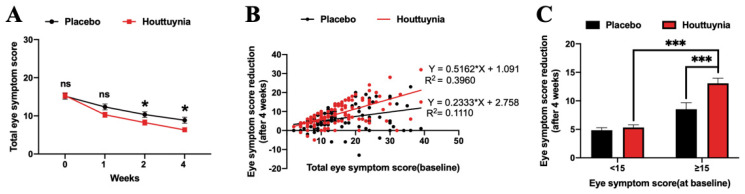
Change in eye symptom score. (**A**) shows eye symptom scores at baseline, one week, two weeks, and four weeks. (**B**) shows the linear correlation between decreases in eye symptom score after four weeks of treatment and scores at baseline. Points represent the values in this figure. The regression line equation and the correlation coefficient R squared value are also shown in this figure. (**C**) highlights the reduction in eye symptom score after four weeks of therapy in scores <15 and ≥15 (at baseline) in two subgroups. Data are presented as mean ± standard error of the mean (SEM). * *p* < 0.05, *** *p* < 0.001. ns, no significance.

Total and individual ocular symptom scores gradually decreased in both the groups in the following three visits (Figure 2A). There was a statistically significant difference between the two groups in the score reduction of 1 week (*p* = 0.0002) and 2 weeks (*p* = 0.0020); results reveal a 6.29 ± 0.55 (*p* < 0.0001) and 9.00 ± 0.61 (*p* < 0.0001) reduction in total symptom scores after four weeks of atomization treatment compared with baseline in the placebo and *Houttuynia* groups, respectively, as well as a statistically significant difference between the two (*p* = 0.0018) (Table 1). Results for each individual symptom score show that values for the *Houttuynia* group dropped more than in the placebo group, while reduced values for eye dryness, foreign body sensation, and photophobia were statistically different between the two groups (Table S4).

Figure 2B reveals a positive linear correlation between eye symptom score reduction after four weeks of treatment and scores at baseline in both groups. Correlation coefficient (r) values were 0.3331 (*p* = 0.0002) and 0.6293 (*p* < 0.0001) in the placebo and *Houttuynia* groups, respectively. We then divided patients into two groups according to the severity of their eye symptoms at baseline (Figure 2C); results show that the reductions in scores after four weeks of therapy in scores < 15 of placebo and *Houttuynia* per group were 4.85 ± 0.45 and 5.32 ± 0.47, respectively, and that there were no statistical differences, while for score ≥ 15, the reductions of placebo and *Houttuynia* group were 8.53 ± 1.14 and 13.07 ± 0.90, respectively. A significant statistical difference was seen between these two groups (95% CI, range between −7.38 and −1.70; *p* = 0.0003) with an interaction *p* value = 0.0057 (Table 2). These results demonstrate that the group with symptom scores ≥ 15 compared to scores < 15 improved more significantly when treated with *Houttuynia* eye drops.

**Figure 3:**
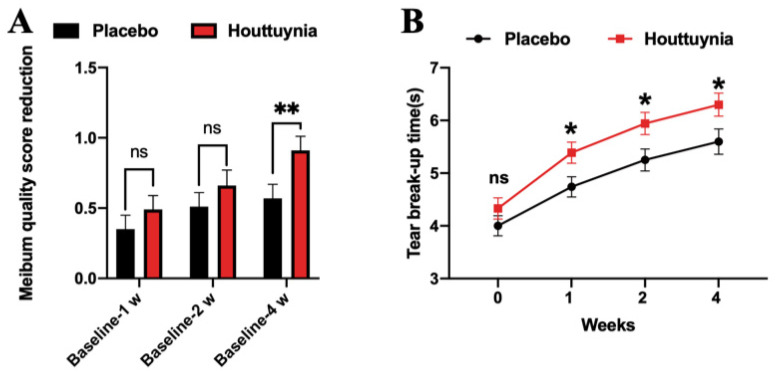
Change in meibum quality and tear break-up time. (**A**) shows the meibum quality score reduction values after one week, two weeks, and four weeks compared with the baseline. (**B**) shows tear break-up time at baseline, one week, two weeks, and four weeks. Data are presented as mean ± SEM. * *p* < 0.05, ** *p* < 0.01, ns, no significance.

Results show that both meibum quality and TBUT also improved in both groups (Figure 3). Changes in these two indicators after therapy were also significantly statistically different in the two groups compared with the baseline (*p* < 0.0001) (Table 1). Meibum quality scores decreased after therapy; these reductions were 0.57 ± 0.10 and 0.91 ± 0.10 in the placebo and *Houttuynia* groups after four weeks. There were statistical differences between the Houttuynia and placebo groups in the meibum quality score or the reduction value of the score after four weeks of treatment (*p* = 0.0032/0.0091) (Table 1). Patients were divided into four groups on the basis of meibum quality scores at baseline. Results show that the fall in the *Houttuynia* group was higher amongst each subgroup than in the placebo set (*p* value for interaction = 0.0282) (Table 2). Values for TBUT in the *Houttuynia* group were always higher than those in the placebo group after treatment over different lengths of time. Indeed, there was a statistically significant difference between the two groups in the TBUT of 1 week (*p* = 0.0300) and 2 weeks (*p* = 0.0199). After four weeks of treatment, TBUT was 5.60 ± 0.24 s and 6.30 ± 0.22 s (*p* = 0.0172) in the placebo and *Houttuynia* groups, respectively.

#### 3.1.2. Secondary Outcomes

**Figure 4:**
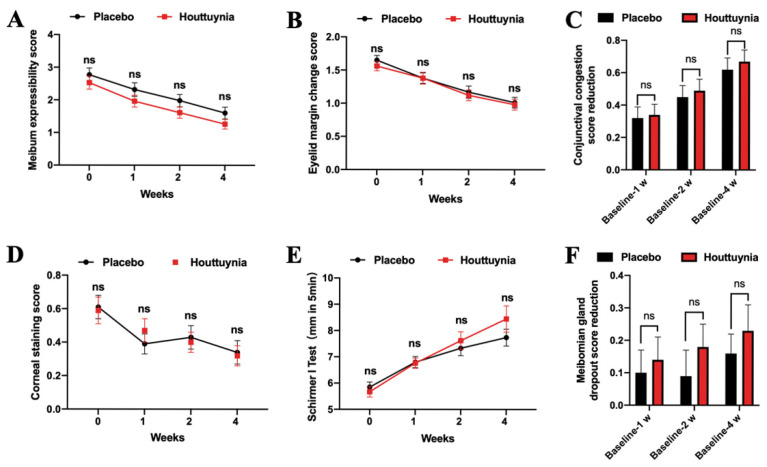
Changes in secondary outcome measures. (**A**–**E**) illustrate meibum expressibility scores, eyelid margin change, corneal staining, and Schirmer I test results, respectively. These data are shown at baseline as well as after one week, two weeks, and four weeks. (**C**,**F**) show reduction values for conjunctiva congestion and meibomian gland dropout scores after one week, two weeks, and four weeks compared with the baseline. Data at each point are presented as mean ± SEM. ns, no significance.

Secondary outcomes in the two groups also improved after four weeks of treatment (Figure 4A–F). Meibum expressibility scores in the *Houttuynia* group subsequent to treatment remained continuously low compared to the placebo group and there were no statistical differences. Patients were then divided into four groups according to their middle meibomian gland expression scores at baseline; this enabled us to demonstrate that decreases in the *Houttuynia* group among each subgroup were higher than those in the placebo group and there were statistical differences for interactions (*p* = 0.0051) (Table 2).

Eyelid margin change scores in the *Houttuynia* group were also continuously low compared to the placebo group, although there were no statistical differences. A positive correlation was found between decreases in eyelid margin change scores after four weeks of treatment and these scores at baseline in the two groups. The r-values in this case were 0.7707 (*p* = 0.1272) and 0.9890 (*p* = 0.0014) in the placebo and *Houttuynia* groups, respectively (Table S5). The eyelid margin score reduction of the *Houttuynia* group was more overall than that of the placebo group in each subgroup case, encapsulating a statistically significant interaction difference (*p* = 0.0158) (Table 2).

Results show that conjunctival congestion improved following atomization treatment. Decreased values of the placebo and *Houttuynia* groups were 0.61 ± 0.07 (*p* < 0.0001) and 0.63 ± 0.06 (*p* < 0.0001) in one subgroup (score = 1 at baseline) and 0.86 ± 0.17 (*p* < 0.0001) and 1.05 ± 0.20 (*p* < 0.0001) in another subgroup (score = 2 at baseline) (*p* value for interaction = 0.6094) (Table 2). The corneal staining score also reduced after treatment: values were 1.07 ± 0.37 and 1.13 ± 0.48 in the placebo and *Houttuynia* groups, respectively. There was no statistical difference in the subgroup at the last visit (corneal staining score > 1 at baseline) (Table 2). Volumes for the Schirmer I test were 7.74 ± 0.32 mm/5 min and 8.45 ± 0.50 mm/5 min (*p* value for interaction = 0.6298) in the placebo and *Houttuynia* groups, respectively (Table 1). Meibomian gland dropout score reduction gradually increased; score reduction in the *Houttuynia* group was higher than in the placebo group and there was no statistical difference.

Thus, taking all these data together, they suggest that treatment with *Houttuynia* was more effective than treatment with placebo.

### 3.2. Safety Assessments

Visual acuity and IOP values for the two groups remained basically unchanged following medication. Patients in one center (Peking Union Medical College Hospital) were assessed for blood/urine routines and liver/kidney functions but all indicators remained basically unchanged following medication. Although some patients exhibited abnormal indicators after medication, there were no clinically significant differences in index values and indicators returned to normal in the follow-up.

The data presented in Table S6 reveal that seven adverse event cases were seen throughout the test. Four of these were in the placebo group (i.e., animal wool allergy, upper respiratory tract infection, blurred vision, and allergic conjunctivitis), and the incidence rate of these events was 2.50%, while three further cases were in the *Houttuynia* group (i.e., upper respiratory tract infection, conjunctivitis, and allergic conjunctivitis). The incidence rate for the cases was 2.54% and data show no statistically significant difference between the two groups. Two subjects in the placebo group and one in the Houttuynia group were withdrawn from the trial due to these adverse events. Indeed, as all adverse event cases either recovered or were relieved, we consider these irrelevant to the experimental treatment.

## 4. Discussion

The novel results of this strictly randomized, double-blinded, placebo-controlled clinical trial regarding the use of ultrasound-atomized *Houttuynia* eye drops for the treatment of MGD-related DED demonstrate that both the symptoms and signs of this condition are statistically significantly ameliorated from the baseline before treatment. Primary efficacy indicators were significantly enhanced compared with the placebo control group, and differences between the two groups were statistically significant. The specific clinical data were as follows: patients’ eye symptom scores decreased by an average of 9 points, meibum quality decreased by nearly 1 point (indicating an improvement of nearly a grade), and TBUT increased to an average of 6.3 s after 4 weeks of treatment. The results of our subgroup analysis also show that the more severe the symptoms and the more turbid the nature of meibomian gland secretions, the better the therapeutic effect of *Houttuynia* eye drops will be. These results suggested that ultrasonically atomized *Houttuynia* eye drops had a clear positive effect on the treatment of MGD-related DED. This therapy can obviously relieve eye-related symptoms, improve meibum quality, and prolong tear break-up time in the clinic.

Inflammation is the main pathological manifestation of DED. This means that anti-inflammatory treatment is the most commonly used therapy for this condition. Inhibiting inflammation can stabilize the tear film and improve the clinical symptoms and signs of DED; indeed, the ultrasonic atomization of *Houttuynia* eye drops can improve both the symptoms and signs of DED, perhaps mainly related to the anti-inflammatory and antibacterial effects of this plant [11,12,13]. MGD-related DED is characterized by terminal duct obstruction and/or qualitative/quantitative changes in glandular secretions. This is because, on one hand, lipid discharge into the tear film is reduced, causing excessive tear evaporation and tear hyperosmolarity. Tear hyperosmolarity, therefore, also stimulates a cascade in the epithelial cells of the ocular surface, involving MAPK and NF-κB signaling pathways as well as the generation of inflammatory cytokines (i.e., interleukin-1 (IL-1α; IL-1β)), tumor necrosis factor-α (TNF-α)), and proteases. These activate and recruit inflammatory cells, induce inflammatory cascade reactions, and promote inflammation of the meibomian gland, eyelid margin, and ocular surface [25]. On the other hand, meibum stasis inside the gland can promote bacterial proliferation (including *Staphylococcus aureus* and other species); growth of these microorganisms enhances the production of lipid-degrading lipases and esterases that decompose the meibum into toxic mediators including free fatty acids. These toxic mediators increase meibum viscosity and melting temperature and result in tear film instability. Inflammation and the release of inflammatory cytokines can also occur; these phenomena aggravate the destruction of meibomian glands and, again, lead to a vicious circle [7]. The plant *H. cordata* is a traditional Chinese medicine therapy and has been shown to have significant anti-inflammatory and antibacterial effects. Indeed, results show that extracts from this species have anti-inflammatory effects on various cell and animal models. The main anti-inflammatory mechanism in this case acts to reduce the level of inflammation-related cytokines (i.e., TNF-α and IL-1β) and chemokines by inhibiting the NF-κB/MAPK pathway. It is clear that the NF-κB/MAPK pathway is one transduction inflammation pathway caused by MGD-related DED. One in vitro bacteriostatic test showed that *H. cordata* exerts an obvious inhibitory effect on catarrhal bacteria, *S. aureus*, influenza bacillus, and pneumococcus [11,26]. Clinical observations have also shown that this treatment can inhibit bacteria on the ocular surface and can therefore be useful for bacterial conjunctivitis therapy. *Houttuynia* eye drops can inhibit bacterial growth on ocular surfaces and meibomian glands, thereby improving meibum quality.

The results of this study show that patients in the placebo group exhibited improved symptoms as well as all signs after four weeks of treatment. These data were statistically different from those seen before treatment and are mainly related to the ultrasonic atomization treatment method. A number of clinical studies have shown that this method using different liquid can improve meibum expressibility, stabilize the tear film, increase fluid, and mitigate the symptoms and signs of DED [10,11,12]. The placebo in this study was a sodium chloride solution. A previous study showed that eye atomization treatment with saline alone can also improve the symptoms and signs (including TBUT, corneal fluorescent staining, and Schirmer I test) of dry eye patients. Compared with the artificial tear (0.1% sodium hyaluronate eye fluid of Shentian Pharmaceutical Co., LTD.) group, the therapeutic effect of the saline atomization group was significantly better (*p* < 0.05) [12]. Ultrasonic atomization makes droplets uniformly, continuously, and comprehensively act on the cornea, conjunctiva, and eyelid, maximizes the contact area between an eye and liquid, and therefore speeds up drug absorption [10]. Therefore, in this study, there was no statistical difference in the change values of TBUT and tear secretion after 4 weeks of treatment, which may be related to the significant improvement effect of ultrasonic atomization on DED. This study has further clarified the effectiveness of this treatment.

Although the experimental group did not exhibit any statistical differences in terms of secondary efficacy indicators in comparison with the placebo group, data on the improvement of symptoms and signs in this former group were better than those in the latter. In addition, post hoc subgrouping analysis was conducted. Due to the limited number of samples, though the subgroup analysis cannot draw accurate conclusions, it can provide potential directions and ideas for the study. Thus, subsequent to grouping secondary indicators according to baseline severity, the data in this study show a statistical difference between the two groups in terms of meibum expressibility and eyelid margin change. This may suggest that patients with more severe meibomian gland blockage and worse eyelid margin state will experience more marked improvements as a result of *Houttuynia* eye drops when compared to the placebo set. This might be due to the therapeutic effects of the ultrasonic atomization method. No statistical differences were seen between conjunctival congestion and corneal fluorescent staining; this may be related to the low occurrence of severe conjunctival congestion and corneal fluorescent staining in the included patients. A further possibility might be that treatment times were not long enough and sample sizes were not big enough; larger samples and longer-term research projects will therefore be needed.

The results of visual acuity, IOP, and laboratory examinations after four weeks of treatment were basically unchanged and there were no adverse events related to the test drug. This shows again that this treatment is safe. Indeed, regarding subjective evaluation results, patients tolerated *Houttuynia* eye drop atomization treatments well, there was little irritation, and individuals were willing to tolerate this approach.

The main limitation of this study was that *Houttuynia* eye drops were not compared with other anti-inflammatory drugs. It is therefore unclear whether, or not, the therapeutic effect of *Houttuynia* eye drops is superior to other anti-inflammatory drugs commonly used in clinical practice. Inflammatory cytokines of tears were not detected during the delivery of this medication, another important indicator that can be used to evaluate eye surface inflammatory state. This is another important indicator that can be used to reflect *Houttuynia* eye drop mechanisms. Another major limitation was that there was missing correction for multiple comparisons. This study tested for multiple hypotheses. The test was considered valid when all three primary indicators were statistically significant. The three indices are independent of each other, and the results of the individual tests are essential, hence we made the decision not to adjust *p* values.

We have shown that treating patients with mild to moderate MGD-related DED for one month via the ultrasonic atomization of *Houttuynia* eye drops can significantly ameliorate the symptoms of eye discomfort, improve meibomian gland function, and stabilize tear films. The results of this study show that this treatment is safe.

## Figures and Tables

**Table 1:** Changes in eye symptoms and signs parameters after 4 weeks of treatment.

Variables	Placebo (*N* = 120)				Houttuynia (*N* = 118)				*p* Value for Column Factor (4 Weeks/Change)
	Baseline	After Treatment (4 Weeks)	Change	*p* Value *	Baseline	After Treatment (4 Weeks)	Change	*p* Value *	
**Primary outcome**									
**Eye symptom score**									
Mean ± SEM	15.15 ± 0.78	8.86 ± 0.79	6.29 ± 0.55	**<0.0001**	15.32 ± 0.74	6.32 ± 0.59	9.00 ± 0.61	**<0.0001**	**0.0152/0.0018**
**Meibum quality score**									
Mean ± SEM	0.92 ± 0.08	0.56 ± 0.07	0.57 ± 0.10	**<0.0001**	0.85 ± 0.08	0.31 ± 0.05	0.91 ± 0.10	**<0.0001**	**0.0032/0.0091**
**Tear break-up time(s)**									
Mean ± SEM	4.00 ± 0.19	5.60 ± 0.24	1.61 ± 0.20	**<0.0001**	4.33 ± 0.20	6.30 ± 0.22	1.97 ± 0.17	**<0.0001**	**0.0172/0.1648**
**Secondary outcome**									
**Meibum expressibility score**									
Mean ± SEM	2.77 ± 0.21	1.60 ± 0.19	1.67 ± 0.25	**<0.0001**	2.53 ± 0.20	1.26 ± 0.15	2.06 ± 0.29	**<0.0001**	0.3535/0.1719
**Eyelid margin change score**									
Mean ± SEM	1.65 ± 0.07	1.01 ± 0.08	0.65 ± 0.08	**<0.0001**	1.56 ± 0.07	0.98 ± 0.08	0.59 ± 0.08	**<0.0001**	0.7038/0.5411
**Conjunctival congestion score**									
Mean ± SEM	0.90 ± 0.06	0.43 ± 0.06	0.62 ± 0.07	**<0.0001**	0.89 ± 0.06	0.38 ± 0.05	0.67 ± 0.07	**<0.0001**	0.7089/0.6403
**Corneal staining score**									
Mean ± SEM	0.61 ± 0.07	0.34 ± 0.07	0.54 ± 0.14	**0.0004**	0.59 ± 0.08	0.32 ± 0.06	0.54 ± 0.12	**<0.0001**	0.9831/0.8253
**Schirmer I test**									
Mean ± SEM	5.85 ± 0.19	7.74 ± 0.32	1.88 ± 0.32	**<0.0001**	5.67 ± 0.20	8.45 ± 0.50	2.77 ± 0.52	**<0.0001**	0.6298/0.7769
**Meibomian gland dropout score**									
Mean ± SEM	0.56 ± 0.07	0.50 ± 0.07	0.16 ± 0.06	**0.0313**	0.51 ± 0.06	0.40 ± 0.06	0.23 ± 0.08	**0.0117**	0.2744/0.3069

Change value = post-treatment value—pre-treatment value (tear break-up time, Schirmer I test); change value = pre-treatment value—post-treatment value (eye symptom score, meibum quality score, meibum expressibility score, eyelid margin change score, conjunctival congestion score, corneal staining score, and meibomian gland dropout score). *p* Value * shows the statistical difference that compared baseline and post-treatment for the same group. *p* Value for Column Factor shows the statistical difference that compared the placebo group and Houttuynia group. Abbreviations: N, number; SEM, standard error of the mean.

**Table 2:** Subgroup analysis.

Variables	Placebo		Houttuynia		Mean Difference (95%CI)	*p* Value for Interaction
	*N*	Mean ± SEM	*N*	Mean ± SEM		
**After 4-week therapy**						
**Primary outcome**						
**Eye symptom score reduction**						**0.0057**
Score < 15 (at baseline)	73	4.85 ± 0.45	62	5.32 ± 0.47	−0.47 (−2.96 to 2.01)	
Score ≥ 15 (at baseline)	47	8.53 ± 1.14	56	13.07 ± 0.90	**−4.54 (−7.38 to −1.70)**	
**Meibum quality score reduction**						**0.0282**
Score = 0 (at baseline)	49	−0.08 ± 0.04	49	−0.02 ± 0.02	−0.06 (−0.41 to 0.29)	
Score = 1 (at baseline)	40	0.35 ± 0.08	43	0.54 ± 0.09	0.18 (−0.57 to 0.20)	
Score = 2 (at baseline)	23	0.96 ± 0.18	21	1.62 ± 0.13	**−0.66 (−1.19 to −0.14)**	
Score = 3 (at baseline)	8	1.38 ± 0.46	5	1.60 ± 0.51	−0.23 (−1.22 to 0.77)	
**Secondary outcome**						
**Middle meibomian gland expression score reduction**						**0.0051**
Score = 0 (at baseline)	42	−0.12 ± 0.06	49	−0.08 ± 0.05	−0.04 (−0.43 to 0.36)	
Score = 1 (at baseline)	52	0.52 ± 0.08	42	0.57 ± 0.10	−0.05 (0.44 to 0.34)	
Score = 2 (at baseline)	23	1.00 ± 0.18	25	1.24 ± 0.18	−0.24 (−0.79 to −0.31)	
Score = 3 (at baseline)	3	1.00 ± 0.58	2	3.00 ± 0.00	**−2.00 (−3.72 to −0.28)**	
**Eyelid margin change score reduction**						**0.0158**
Score = 0 (at baseline)	2	−1.00 ± 1.00	3	0.00 ± 0.00	−1.00 (−3.35 to 1.35)	
Score = 1 (at baseline)	57	0.25 ± 0.08	61	0.38 ± 0.09	−0.13 (0.61 to 0.34)	
Score = 2 (at baseline)	46	1.11 ± 0.11	42	1.00 ± 0.13	−0.11 (−0.44 to 0.66)	
Score = 3 (at baseline)	11	1.64 ± 0.24	9	1.78 ± 0.40	−0.14 (−1.30 to 1.02)	
Score = 4 (at baseline)	4	0.75 ± 0.75	3	2.67 ± 1.33	−1.92 (−3.89 to 0.05)	
**Conjunctival congestion score reduction**						0.6094
Score = 0 (at baseline)	33	−0.06 ± 0.04	32	−0.06 ± 0.04	0.00 (−0.39 to 0.39)	
Score = 1 (at baseline)	66	0.61 ± 0.07	67	0.63 ± 0.06	−0.02 (−0.29 to 0.25)	
Score = 2 (at baseline)	21	0.86 ± 0.17	19	1.05 ± 0.20	−0.20 (−0.69 to 0.30)	
**Corneal staining score reduction**						0.9723
Score = 0 (at baseline)	65	−0.11 ± 0.05	65	−0.12 ± 0.04	0.02 (−0.32 to 0.35)	
Score = 1 (at baseline)	41	0.56 ± 0.11	45	0.58 ± 0.10	0.02 (−0.39 to 0.42)	
Score > 1 (at baseline)	14	1.07 ± 0.37	8	1.13 ± 0.48	−0.05 (−0.89 to 0.78)	

Score reduction value = pre-treatment value—post-treatment value. Abbreviations: N, number; SEM, standard error of the mean; CI, confidence interval.

## Supplementary Materials

The following are available online at https://www.mdpi.com/2077-0383/9/12/4022/s1, Figure S1: The ultrasonic nebulizer, Table S1: Specific distribution of cases at each center, Table S2: Ocular symptom rating scale, Table S3: Characteristics of the patients at baseline, Table S4: Changes in individual eye symptom after 4-week treatment, Table S5: Correlation between indicator change and that at baseline, Table S6: Adverse events during the trial.

## Abbreviations

MGD (meibomian gland dysfunction), DED (dry eye disease), TBUT (tear break-up time), AE (adverse event), SEM (standard error of the mean), EDE (evaporative dry eye), CATCM (China Association of Traditional Chinese Medicine), IOP (intraocular pressure), ITT (intention to treat), CI (confidence interval).
