# Supplementary material for: Efficacy and Safety of Houttuynia Eye Drops Atomization Treatment for Meibomian Gland Dysfunction-Related Dry Eye Disease: A Randomized, Double-Blinded, Placebo-Controlled Clinical Trial
Source: J Clin Med. 2020 Dec 12;9(12):4022. doi: 10.3390/jcm9124022 (PMC7763017; doi:10.3390/jcm9124022)
Supplement: Supplementary file 1 [file jcm-09-04022-s001.pdf]

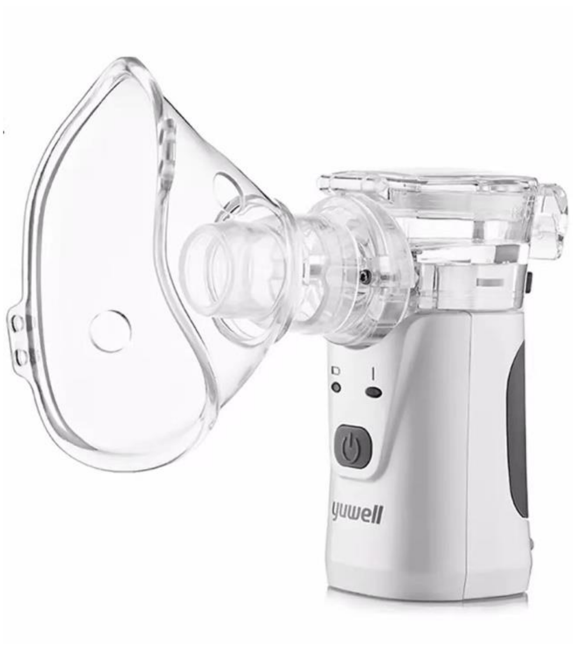

Figure S1. The ultrasonic nebulizer. The ultrasonic nebulizer (product number: HL100A, Yuwell, Jiangsu, China) was shown in Figure S1. It is a handheld atomizer, weighing about 85g, the average diameter of atomized droplets is about 3.5 μm, and the proportion of droplets below 5μm > 60%. Flow rate ≥ 0.2ml /min.

Table S1. Specific distribution of cases at each center.

	Placebo (Number)		Houttuynia (Number)		Total (Number)	
**Center**	**Enrollment**	**Analyzed**	**Enrollment**	**Analyzed**	**Enrollment**	**Analyzed**
1	12	12	12	12	24	24
2	18	18	18	17	36	35
3	12	12	12	12	24	24
4	20	20	20	20	40	40
5	12	12	12	12	24	24
6	12	12	12	12	24	24
7	18	18	18	17	36	35
8	16	16	16	16	32	32
**Total**	120	120	120	118	240	238

1, Xiamen University affiliated Xiamen Eye Center, Xiamen, Fujian, China.

2, China-Japan Friendship Hospital, Beijing, China.

3, Capital Medical University affiliated Beijing Tongren Hospital, Beijing, China.

4, Peking Union Medical College Hospital, Beijing, China.

5, Shandong University of Traditional Chinese medicine affiliated Eye Hospital, Jinan, Shandong, China.

6, Hebei Eye Hospital, Xingtai, Hebei, China.

7, Shenzhen Eye Hospital, Shenzhen, China.

8, Xi'an First Hospital, Xi’an, Shanxi, China.

Table S2. Ocular symptom rating scale

Symptom	No (0)	Occasionally (1,2,3)	Half the time (4,5,6)	Most of the time (7,8,9)	Full time (10)
Dryness of eye					
Foreign body sensation of eye					
Eye pain					
Photophobia					
Secretion on eyelashes in the morning, difficulty in opening eyes					
Fluctuation of vision					

Table S3. Characteristics of the patients at baseline.

Variables	Placebo (N=120)	Houttuynia (N=118)	Statistics	*P* Value
**Demographics**				
**Sex**				
Male n(%)	30(25.00)	28(23.73)	χ^2=0.0522	0.8193
Female n(%)	90(75.00)	90(76.27)		
**Age(year)**				
Mean±SEM	38.30±1.08	38.74±1.12	Z=0.1836	0.8543
**Vision**				
**Uncorrected visual acuity**	N=51	N=40		
Mean±SEM	0.94±0.04	0.92±0.02	Z=-1.0422	0.2973
**Corrected visual acuity**	N=69	N=78		
Mean±SEM	0.81±0.03	0.83±0.03	Z=-0.5507	0.5819
**Primary outcome**				
**Eye symptom score**				
Mean±SEM	15.15±0.78	15.32±0.74	Z=0.3912	0.6956
**Meibum quality score**				
0 n(%)	49(40.83)	49(41.53)	Z=-0.4443	0.6568
1 n(%)	40(33.33)	43(36.44)		
2 n(%)	23(19.17)	21(17.80)		
3 n(%)	8(6.67)	5(4.24)		
**Tear breakup time(s)**				
Mean±SEM	4.00±0.19	4.33±0.20	Z=1.2301	0.2187
**Secondary outcome**				
**Meibum expressibility score**				
Mean±SEM	2.77±0.21	2.53±0.20	Z=-0.6991	0.4845
**Eyelid margin change score**				
Mean±SEM	1.65±0.07	1.56±0.07	Z=-0.8769	0.3805
**Conjunctival congestion score**				
0 n(%)	33(27.50)	32(27.12)	Z=-0.1031	0.9179
1 n(%)	66(55.00)	67(56.78)		

2 n(%)	21(17.50)	19(16.10)		
3 n(%)	0(0.00)	0(0.00)		
**Corneal staining score**				
Mean±SEM	0.61±0.07	0.59±0.08	*Z*=-0.3292	0.7420
**Schirmer I test**				
Mean±SEM	5.85±0.19	5.67±0.20	*Z*=-0.7671	0.4430
**Meibomian gland dropout score**				
0 n(%)	74(61.67)	71(60.17)	*Z*=-0.1906	0.8488
1 n(%)	25(20.83)	34(28.81)		
2 n(%)	21(17.50)	13(11.02)		
3 n(%)	0(0.00)	0(0.00)		

If the patient wore glasses, the corrected visual acuity should be recorded, without recording the uncorrected visual acuity. If the patient wore no glasses, the uncorrected visual acuity was recorded.

Abbreviations: N, number; SEM, standard error of the mean.

Table S4. Changes in individual eye symptom after 4-week treatment.

Variables	Placebo (N=120)				Houttuynia (N=118)				*P* Value for column factor (change)
	Baseline	After treatment (4 weeks)	Change	*P* Value*	Baseline	After treatment (4 weeks)	Change	*P* Value*	
**Eye symptom score**									
**Dryness of eye**									
Mean±SEM	5.33±0.24	3.38±0.23	1.96±0.23	<0.0001	5.42±0.25	2.45±0.22	3.03±0.24	<0.0001	**0.0019**
**Foreign body sensation of eye**									
Mean±SEM	3.37±0.24	2.06±0.23	1.44±0.22	<0.0001	3.40±0.26	1.56±0.21	2.11±0.24	<0.0001	**0.0496**
**Eye pain**									
Mean±SEM	1.74±0.26	0.83±0.19	1.62±0.30	<0.0001	1.77±0.24	0.63±0.15	1.88±0.22	<0.0001	0.4977
**Photophobia**									
Mean±SEM	1.73±0.31	1.13±0.28	1.18±0.23	<0.0001	1.75±0.31	0.76±0.23	1.87±0.24	<0.0001	**0.0199**
**Secretion on eyelashes in the morning, difficulty in opening eyes**									
Mean±SEM	1.88±0.33	1.07±0.24	1.53±0.31	<0.0001	1.69±0.29	0.59±0.16	2.06±0.29	<0.0001	0.0882
**Fluctuation of vision**									
Mean±SEM	1.11±0.27	0.40±0.21	1.85±0.29	<0.0001	1.29±0.27	0.33±0.11	2.17±0.28	<0.0001	1.0000

Change value = pre-treatment value - post-treatment value.

P Value* showed the statistical difference that compared baseline and post-treatment for the same group.

P Value for column factor showed the statistical difference that compared the placebo group and Houttuynia group.

Abbreviations: N, number; SEM, standard error of the mean.

Table S5. Correlation between indicator change and that at baseline.

Correlation	Placebo			Houttuynia		
	r	95% CI	*P* value	r	95% CI	*P* value
Reduction of eye symptom score after 4 weeks and eye symptom score at baseline	0.3331	0.1636 to 0.4835	**0.0002**	0.6293	0.5061 to 0.7273	**<0.0001**
Reduction of eyelid margin change after 4 weeks and eyelid margin change score at baseline	0.7707	-0.3487 to 0.9839	0.1272	0.9890	0.8381 to 0.9993	**0.0014**

Abbreviations and Acronyms: CI, confidence interval (CI).

Table S6. Adverse events during the trial.

Group	Number of cases (incidence)	Adverse event	Severity	Effects on experimental drugs	Judgment of relevance to experimental drugs	Adverse event outcome	Whether to withdraw from the trial due to this adverse event
**Placebo**	4(2.50%)	Animal hair allergy	Moderate	Intermittent use	Might be irrelevant	Recover	No
		Upper respiratory infection	Mild	No effect	Might be irrelevant	Recover	No
		Blurred vision	Mild	Stop use	Might be irrelevant	Relieve	Yes
		Allergic conjunctivitis	Mild	Stop use	Might be irrelevant	Recover	Yes
**Houttuynia**	3(2.54%)	Upper respiratory infection	Mild	No effect	Might be irrelevant	Recover	No
		Conjunctivitis	Mild	No effect	Might be irrelevant	Recover	No
		Allergic conjunctivitis	Mild	Stop use	Might be irrelevant	Recover	Yes
